# Sense of control and diabetes mellitus among U.S. adults: A cross-sectional analysis

**DOI:** 10.1186/1751-0759-1-19

**Published:** 2007-10-30

**Authors:** Kathryn M Cardarelli, Sally W Vernon, Elizabeth R Baumler, Susan Tortolero, M David Low

**Affiliations:** 1 Department of Epidemiology, University of North Texas Health Science Center, 3500 Camp Bowie Boulevard, Fort Worth, TX 76107, USA; 2 Division of Behavioral Sciences and Health Promotion, University of Texas Health Science Center, 1200 Herman Pressler Houston, TX 77030, USA; 3 Center for Health Promotion and Prevention Research, University of Texas Health Science Center, 1200 Herman Pressler Houston, TX 77030, USA; 4 Division of Management, Policy, and Community Health, University of Texas Health Science Center, 1200 Herman Pressler Houston, TX 77030, USA

## Abstract

**Background:**

Little is known about the influence of psychosocial factors on diabetes mellitus. The aim of this study was to improve understanding of the association between two psychosocial factors- sense of control and social support- and diabetes mellitus.

**Methods:**

The authors analyzed data from 2,592 U.S. households in the 1995 survey of the Aging, Status, and the Sense of Control study. Logistic regression analyses were conducted to examine whether sense of personal control and social support were associated with DM and whether gender, race, and Hispanic ethnicity modified these associations.

**Results:**

After adjusting for age, obesity, and socioeconomic position, a one point increase in sense of control (i.e., a stronger sense of control) was associated a significant reduction in risk of diabetes mellitus (odds ratio = 0.67, 95% confidence interval: 0.47, 0.95). A weak social support system was associated with a non-significant risk of diabetes (odds ratio = 1.32, 95% confidence interval: 0.93, 1.89). No effect modification was detected.

**Conclusion:**

Sense of control deserves greater attention as a predictor of diabetes mellitus. Further studies of the contribution of psychosocial factors to diabetes mellitus should assess the temporal nature of this relationship.

## Background

Over the past thirty years, psychosocial variables have emerged as important risk factors for cardiovascular disease [1] but similar attention has not been paid to their potential contribution to diabetes mellitus (DM). Previous research of psychosocial characteristics related to DM has focused largely on their role in the management of the illness [2]. Factors such as anger, hostility, depression, lack of social support, and locus of control have been associated with coronary heart disease incidence and mortality [3-6]. Because type 2 DM shares many risk factors with cardiovascular impairment, it seems plausible that some of these same psychosocial factors may impact one's risk for developing DM.

Type 2 DM, which accounts for 90%–95% of the total DM prevalence in the United States, is associated with obesity and physical inactivity [7], largely preventable risk factors, which have been linked to a sense of control [8,9] and to social support [9,10]. Sense of control is the belief that one can and does master, control, and shape one's own life, and is related to self-efficacy and locus of control [11]. Sense of control may provide empowerment to synthesize health-promoting behaviors into a coherent lifestyle, thereby reducing one's risk for a number of diseases [11]. Social support is the perception of existing within social embeddedness; that is, having friends or family who may provide comfort and assistance [12]. Social support may improve psychological well-being and is directly associated with physical health [13]. There is a large body of evidence that documents lower risk of depression and psychological distress for individuals with a high degree of social support [14]. Likewise, social support has been linked with cardiovascular disease [15-18].

In the present study, we examined whether a strong sense of personal control and strong social support were associated with a reduced self-reported prevalence of DM in a U.S. population. In addition, we examined whether these relationships were modified by sociodemographic characteristics including gender, race, or Hispanic ethnicity considering DM affects a disproportionate number of minorities in the U.S.

## Methods

This study used data from the Aging, Status, and Sense of Control (ASOC) survey, a representative national telephone survey of 2,592 English-speaking respondents aged 18 to 95, with an oversample of those aged 60 and older. The ASOC study, conducted in 1995, examined the relationship between age and changes in the sense of control over one's own life. The Survey Research Laboratory of the University of Illinois conducted data collection, and the National Institute on Aging funded the study (R01 AG12393, PI: John Mirowsky). A prescreened random-digit dialing method was used to decrease the probability of contacting a business or nonworking number and to decrease the standard errors compared with the Mitofsky-Waksberg method, while producing a sample with similar demographic profile [19].

English-speaking persons age 18 or older were eligible to participate in the survey, and two subsamples were devised to produce an 80% oversample of persons age 60 or older. In the main sample, the adult with the most recent birthday was selected as a participant. In the other sample, the person aged 60 years or older with the most recent birthday was selected to participate. Interviews were completed with 71.6% of contacted, eligible persons, resulting in 2,592 participants ranging in age from 18 to 95. Respondents were asked about their physical health, including activities of daily living; mental health, including anxiety and enjoyment of life; health behaviors; use of health care services; sense of control over their lives; social support and participation in community activities; and social hardship, including assault and extended unemployment. Table 1 demonstrates the comparability of the ASOC study population with the general U.S. population, using 1995 demographic statistics from the U.S. Census Bureau [20].

**Table 1:** Comparability of Aging, Status, and Sense of Control Study, 1994–1995, population with 1995 general U.S. population, age 18 years and older

**Characteristic**	**ASOC**	**U.S.**
Female	56%	51%
White	85%	83%
Married	56%	55%
Household size^a^	2.7	2.6
Household income^a^	$43,949	$41,285

^a^Presented as mean value

Respondents who answered the following question with an affirmative response were considered diabetics: "Have you ever been diagnosed or told by a doctor that you have diabetes?" Types 1 and 2 DM were not distinguished and additionally age of onset was not ascertained. Social support was conceptually defined as resources provided by other persons [21], including emotional and instrumental support. Emotional support includes the things that individuals do to make a person feel connected, loved, and cared for, while instrumental support refers to the type of assistance that others provide [22]. To measure emotional support, respondents were asked, "How much do you agree with the statements: 'I have someone I can turn to for support and understanding when things get rough,' and 'I have someone I can really talk to.' To measure instrumental support, respondents were asked, "How much do you agree with the statements, 'I have someone who would help me out with things, like give me a ride, watch the kids or house, or fix something,' and 'I have someone who would take care of me if I were sick." Responses were coded 1= strongly agree, 2 = agree, 3 = disagree, 4 = strongly disagree; and the mean response served as an index of social support. A low score indicates a high degree of social support. This index was found to have a coefficient alpha in the ASOC study population of 0.85 [23].

A personal control scale created by Mirowsky & Ross [24] was used to measure sense of control. Responses to the following four perceived control questions were coded 2 = strongly agree, 1 = agree, 0 = neutral, -1 = disagree, -2 = strongly disagree:

### Control over good

(1) "I am responsible for my own successes"

(2) "I can do just about anything I really set my mind to"

### Control over bad

(3) "My misfortunes are the result of mistakes I have made"

(4) "I am responsible for my failures"

Responses to the following four perceived powerlessness questions are coded 2 = strongly disagree, 1 = disagree, 0 = neutral, -1 = agree, -2 = strongly agree.

### Powerlessness over good

(1) "The really good things that happen to me are mostly luck"

(2) "There's no sense planning a lot–if something good is going to happen, it will"

### Powerlessness over bad

(3) "Most of my problems are due to bad breaks"

(4) "I have little control over the bad things that happen to me"

The sense of control scale was calculated as a mean score of the responses to these eight questions, with a high score indicating a strong sense of control. This index was found to have a coefficient alpha in the ASOC study population of 0.68 [24].

Self-reported sociodemographic characteristics of respondents included sex, age, race, ethnicity, and socioeconomic position. Age was analyzed as a continuous variable, coded in years. Race was categorized as White, African American, and other (including Asian or Pacific Islander, Native Americans, and those who responded "other"). Ethnicity was dichotomized as either Hispanic or not Hispanic. Because race was not mutually exclusive for Hispanic ethnicity, both race and ethnicity variables were included in the analysis. Body mass index (BMI) was calculated using self-reported height and weight values and were dichotomized into obese (≥ 30 kg/m^2^) or not obese (< 30 kg/m^2^), based on clinical guidelines established by the National Heart, Lung, and Blood Institute [25]. Socioeconomic position was operationalized by education, and ascertained by asking respondents, "What is the highest grade or year in school that you have completed?" This variable was categorized as (1) less than high school, (2) high school completed or some college completed, and (3) college completed or more than a college education.

Because the ASOC survey oversampled adults age 60 years and older by a factor of 1.8, weights were applied to the data. The weight was the inverse of the sampling probability, which was 1 if the respondent was younger than 60 and 1/1.8 if the participant was age 60 or older. In order to retain the correct total number of survey respondents, a final weighting variable was created by using the initial weighting variable divided by the average weight for the entire sample. This weighting factor was utilized in all analyses to bring the study sample into alignment with the target population.

Logistic regression models were created, with DM status regressed on each psychosocial variable separately, adjusted for sex, age, and obesity status. To assess for effect modification, these models were then stratified by race, ethnicity and gender, and interaction terms were also created. Odds ratios and 95% confidence intervals were calculated for each factor.

The software program SPSS version 14.0 was used to manage data and conduct the analyses. The type I error rate was set at the α =.05 level. This study was approved by the Institutional Review Board at the University of Texas Health Science Center at Houston.

## Results

Five percent of the study population, or 131 participants, reported a DM diagnosis. DM was more prevalent in African Americans, obese participants, respondents over the age of 60, and those who were either disabled or retired from employment (Table 2). A higher sense of control and a stronger social support system were inversely associated with DM status.

**Table 2:** Characteristics of the study group, Aging, Status, and Sense of Control Study, 1994–1995

**Characteristic**	**No. in sample**	**% of total population**	**% diabetics in each category**	**p-value**^a^
Sex				
Male	1099	42.4	4.5	.22
Female	1491	57.5	5.5	
Race				
White	2192	84.5	4.6	.001
African American	195	7.5	11.3	
Asian or Pacific Islander	42	1.6	2.3	
Native American	37	1.4	5.4	
Other	124	4.8	3.1	
Ethnicity				
Hispanic	147	5.7	4.1	.58
Not Hispanic	2431	93.8	5.1	
Missing	14	0.5		
Age, in years				
18–39	969	37.4	1.0	<.001
40–59	848	32.7	4.6	
60 +	755	29.1	11.1	
Missing	20	0.8		
Education				
Less than high school	330	12.7	10.7	<.001
High school degree or some college	1556	60.0	4.3	
College degree or some graduate/professional school or graduate/professional degree	686	26.4	4.1	
Body Mass Index				
Obese	428	16.5	10.3	<.001
Not obese	2147	82.8	4.1	
Missing	17	0.7		
Social support^b^	1.7	0.01	---	<.001
Sense of control^b^	0.7	0.01	---	<.001

^a^Chi-square test for categorical variables, independent samples t-test for continuous variables

^b^Presented as mean values and standard errors; independent t-test

The odds ratios for the effect of each psychosocial variable regressed on DM status, adjusted for age, obesity status, and socioeconomic position, are shown in Table 3. For every one point increase in the sense of control scale (or a *stronger *sense of control) the odds of having DM were reduced by 33 percent (95% CI: 0.47–0.95). For every one point increase in the social support scale (or a *weaker *social support system), the odds of having DM increased by 32 percent (95% CI: 0.93–1.89), although the CI indicates that this point estimate is imprecise and results are not statistically significant. Stratification by gender, race, and ethnicity showed no evidence of effect modification. Furthermore, interaction terms for these models were not statistically significant (results not shown). We also found no interaction between sense of control and social support.

**Table 3:** Adjusted odds ratios^a ^for psychosocial factors on diabetes mellitus status, Aging, Status, and Sense of Control Study, 1994–1995

	**Sense of Control**		**Social Support**	
**Variable**	**OR**	**95% CI**	**OR**	**95% CI**

Overall model	0.67	0.47, 0.95	1.32	0.93, 1.89
Males	0.56	0.32, 0.99	1.07	0.61, 1.89
Females	0.70	0.44, 1.11	1.51	0.96, 2.39
White	0.81	0.54, 1.20	1.36	0.92, 2.03
African American	0.31	0.09, 0.99	1.18	0.42, 3.29
Other race	0.72	0.11, 4.83	0.75	0.12, 4.88
Hispanic ethnicity	0.31	0.05, 2.09	1.20	0.25, 5.76

^a^Adjusted for age, obesity, and socioeconomic position

## Conclusion

Our findings show that, after adjusting for covariates, having a strong sense of control was significantly protective against DM. Few studies have previously examined these psychosocial variables as independent correlates for DM. More often, psychosocial factors have been investigated among diabetics in the context of self-management. For example, sense of control has been identified as a predictor of metabolic control among diabetics [26,27], as has social support [28]. DM-specific social support and self-efficacy have been shown to correlate with an improved health related quality of life [29]. Among those studies that have considered psychosocial risk factors for DM, one found effort-reward imbalance related to work environments to be associated with type 2 DM incidence among men only [30]. In contrast, another study found an association between both low decision latitude at work and low sense of coherence with type 2 DM only among women [31].

A low sense of control may result in a compromised ability to deal with environmental or psychological stressors [32]. In response to stress, the hypothalamic-pituitary-adrenal (HPA) axis secretes cortisol, an overabundance of which may contribute to insulin resistance [33]. Additionally, persons with a low sense of control may be less likely to engage in healthy behavior.

Limitations of this study should be considered in interpreting the results. People without telephones were not included in the ASOC survey, and it is likely that those individuals would be of low socioeconomic position, which is associated with DM [34]. In a study that examined the potential coverage bias in telephone surveys, investigators who used data from the NHANES III found that those respondents without a telephone were more likely to be both obese and diabetic [35]. This selection bias has the potential to underestimate the association between psychosocial factors and DM.

Measurement of the outcome was based on respondents' self-reports of DM status. In general, the prevalence of DM in the ASOC study population (5%) is similar to estimates based on self-report from both the National Health Interview Survey and the Behavioral Risk Factor Surveillance System [36,37]. Additionally, undiagnosed DM was not assessed. One study that used laboratory testing to verify DM diagnosis found the prevalence of undiagnosed DM to be 2.7% [38]. This bias also has the potential to underestimate the associations of interest if the disease negatively affects one's sense of control or ability to maintain one's social contacts. It was also not possible to distinguish type I and type 2 DM in the ASOC survey. Although they share some risk factors, type 1 and type 2 DM generally have different characteristics and courses. However, because of the low prevalence of type 1 DM in the general U.S. population (roughly 5%–10% of all DM) [7], and the population-based nature of this study sample, the ambiguity of the type of DM is unlikely to bias the point estimates. Finally, because this study utilized cross-sectional data (and prevalence instead of incidence), no determinations of causality are possible. Although it may be reasonable to assume that adverse psychosocial factors are risks for DM, it is also possible that the reverse is true. Future research should include longitudinal studies to assess temporality of the influence of psychosocial factors on the incidence of DM. In addition, future research may focus on diagnostic criteria, as opposed to self-reported prevalence, as evidence for DM. Distinguishing between levels of received and perceived support with incidence of DM might also provide valuable associations.

Over the last twenty years, DM has emerged as an epidemic in the United States [7]. It is estimated that the number of adults in the U.S. with diagnosed DM (including gestational DM) has risen 61% since 1991, and it is further projected to at least double by the year 2050 [7,36]. The observed association of sense of control and DM prevalence in this study is suggestive of the contribution of an adverse psychological environment in understanding the epidemiology of this condition and warrants further investigation. Particular attention in future research should be paid to the temporal nature of these factors and the incidence of type 2 DM.

## Competing interests

The author(s) declare that they have no competing interests.

## Authors' contributions

Dr. Cardarelli conceptualized the study, conducted the data analysis, and led the writing of the article. Dr. Vernon supervised the direction of the study and assisted with writing the article. Dr. Baumler assisted with the data analysis and with writing the article. Dr. Tortolero assisted in the planning of the study and contributed to the preparation of the article. Dr. Low supervised the study and assisted in preparing the article.

## Acknowledgements

This work was partially supported by the J. McDonald Williams Institute. We are grateful for the comments of two anonymous reviewers.
